# Adrenaline for traumatic cardiac arrest: A post hoc analysis of the PARAMEDIC2 trial

**DOI:** 10.1016/j.resplu.2025.100890

**Published:** 2025-02-04

**Authors:** C. Ji, H. Pocock, C.D. Deakin, T. Quinn, J.P. Nolan, N. Rees, K. Charlton, J. Finn, A. Rosser, R. Lall, G.D. Perkins

**Affiliations:** a Warwick Clinical Trials Unit, Warwick Medical School, University of Warwick, Coventry CV4 7AL, UK; b South Central Ambulance Service NHS Foundation Trust, Bicester, UK; c Southampton University Hospital, Southampton, UK; d Welsh Ambulance Service NHS Trust, Denbighshire, UK; e North East Ambulance Service NHS Foundation Trust, Newcastle-upon-Tyne, UK; f Curtin University, Perth, Australia; g West Midlands Ambulance Service University NHS Foundation Trust, Brierley Hill, UK

**Keywords:** Adrenaline, Advanced life support, Drugs, Resuscitation, Trauma, Traumatic cardiac arrest

## Abstract

**Introduction:**

There is controversy about the effectiveness of adrenaline in traumatic cardiac arrest. This study reports the patient characteristics and outcomes of adults with trauma-related out of hospital cardiac arrest treated with adrenaline or placebo.

**Methods:**

This post-hoc, sub-group analysis of the Pre-hospital Randomised Assessment of Adrenaline in Cardiac Arrest-2 (PARAMEDIC-2) trial explored the effect of adrenaline on survival to hospital admission, longer-term survival and neurological outcomes amongst adults with trauma related out of hospital cardiac arrest. Individual patients were randomised through opening a single treatment pack which contained either 10 doses of 1 mg adrenaline or 0.9% saline placebo. Treating clinicians, investigators, outcome assessors and patients were blinded to treatment allocation. The primary outcome was survival to 30 days post cardiac arrest.

**Results:**

123 of 8,014 enrolled patients (1.5%) sustained a traumatic cardiac arrest (66 in the adrenaline arm and 57 in the placebo arm). Three times as many patients were admitted to hospital alive in the adrenaline arm 16/66 (24.2%) compared to 5/56 (8.9%) in the placebo arm, unadjusted odds ratio 3.3 (95% confidence interval 1.1 to 9.6), *p* = 0.03; adjusted odd ratio 5.6 (95% CI 1.6 to 20.4), *p* = 0.009. A single patient, in the adrenaline arm, survived beyond 30 days (1/66 (1.5%) compared to 0/57 (0%)), who also experienced a favourable neurological outcome.

**Conclusion:**

Adrenaline was associated with a trebling of the rate of survival to hospital admission. These data support the use of adrenaline in trauma related out of hospital cardiac arrest.

**Registration:**

ISRCTN73485024.

## Introduction

The pathophysiology and patient characteristics of trauma related cardiac arrest differs from those observed in cardiac arrest secondary to medical causes. Patients are usually younger, have fewer co-morbidities and are more likely to have a potentially reversible cause of cardiac arrest such as a blood loss, hypoxia, tension pneumothorax and cardiac tamponade.^1^ There is controversy as to whether initial treatment should prioritise treating potentially reversible causes of cardiac arrest above cardiopulmonary resuscitation and pharmacological treatments.^2^, ^3^ Given the low incidence of traumatic cardiac arrest and costs of randomised controlled trials it seems unlikely this uncertainty will be resolved by a future randomised trial. Therefore, we undertook a post-hoc analysis of the Pre-hospital Randomised Assessment of Adrenaline in Cardiac Arrest-2 (PARAMEDIC2) trial,^6^ which explored the patient characteristics and outcomes of adults with trauma related cardiac arrest.

## Methods

The PARAMEDIC2 trial was registered prior to enrolment of the first patient at the ISRCTN Registry (registration number: ISRCTN73485024). The protocol was approved by the South Central–Oxford C Research Ethics Committee (reference number (14/SC/0157) and the Medicines and Healthcare Products Regulatory Authority (EudraCT 2014-000792-11). The trial was sponsored by the University of Warwick and funded by the National Institute for Health and Social Care Research, neither of which had any role in the trial design, in the collection or analysis of the data, or in the writing of the manuscript. The trial protocol, statistical analysis plan and the main trial results have been described in detail previously.^4^, ^5^, ^6^

In brief, adults who sustained an out of hospital cardiac arrest, where resuscitation was attempted by National Health Service (NHS) Paramedics, were randomised to receive 1 mg adrenaline every 3–5 min or 0.9% saline. Patients thought to be pregnant, aged < 16 years, to have sustained a cardiac arrest secondary to asthma or anaphylaxis, or had already received adrenaline were excluded. This post-hoc sub-group analysis focused on patients who were assessed as having sustained a cardiac arrest secondary to trauma.

The primary response to traumatic cardiac arrest involved the dispatch of NHS paramedics who were trained in advanced life support, including airway management, defibrillation, drug administration, needle decompression and crystalloid fluid administration. The primary response may be augmented by attendance of a pre-hospital physician and critical care paramedic who could provide advanced interventions such as thoracotomy and drug facilitated intubation. National clinical guidelines based on recommendations from the European Resuscitation Council,^7^ prioritised the treatment of reversible causes (hypovolaemia, oxygenation, tension pneumothorax) alongside standard advanced life support (chest compressions, airway, drugs). Patients were typically transported to a major trauma centre, unless airway or catastrophic haemorrhage could not be safely managed, in which case the patient would be taken to the nearest emergency department.

Randomisation and allocation concealment was achieved by preparing sequentially numbered treatment packs which were identical in appearance and contained either 10 doses of 1 mg adrenaline or 0.9% saline. The randomisation schedule (1:1 ratio, minimisation method) was generated by a computer programme hosted at Warwick Clinical Trials Unit. Individual patients were randomised through opening a single treatment pack. Treating clinicians, investigators, outcome assessors and patients were blinded to treatment allocation.

The primary outcome for the study was survival to 30 days post cardiac arrest. Secondary outcomes included survival and neurological outcomes, (as assessed by the modified Rankin Scale (which ranges from 0 [no symptoms] to 6 [death]) at discharge from hospital, 3 and 6 months.

Analysis was based on the intention to treat principle with patients who were randomised and received the trial intervention being included in the analyses. Data were summarised as means/ standard deviations or medians and interquartile ranges according to whether data were normally distributed. Frequency and percentage were reported for categorical variables. Survival (including sustained ROSC) and neurological outcomes were analysed using fixed-effect regression models with and without adjustment for covariates (see supplemental material). Analyses were summarised using unadjusted and adjusted odds ratios with 95% confidence intervals.

## Results

Amongst the 8,103 patients who were enrolled in the PARAMEDIC2 trial, 8,014 (98.9%) were recruited by the participating ambulance services of whom 123 (1.5%) had sustained a traumatic cardiac arrest and were included in this study (66 (53.7%) in the adrenaline arm and 57 (46.3%) in the placebo arm). Compared to the overall PARAMEDIC2 population,^6^ patients with traumatic cardiac arrest were younger, more likely to be male and present with a non-shockable rhythm. Patient characteristics between those randomised to adrenaline or placebo were broadly similar (see Table 1) as were pre-hospital resuscitation interventions (see Table 2).

**Table 1:** Baseline and event characteristics.

		Adrenaline N = 66	Placebo N = 57
Age (years)	Mean (SD)	52.7 (24.0)	51.4 (21.0)
	Missing	0	1
Sex	Male	46 (69.7%)	46 (80.7%)
	Female	20 (30.3%)	11 (19.3%)
Initial rhythm	VF	3 (4.5%)	4 (7.0%)
	Pulseless VT	1 (1.5%)	0 (0.0%)
	Asystole	39 (59.1%)	35 (61.4%)
	PEA/EMD	17 (25.8%)	15 (26.3%)
	Bradycardia	0 (0.0%)	1 (1.8%)
	AED non-shockable	1 (1.5%)	0 (0.0%)
	AED shockable	0 (0.0%)	0 (0.0%)
	Unobtainable	0 (0.0%)	0 (0.0%)
	Unknown	5 (7.6%)	2 (3.5%)
Initial rhythm	Shockable rhythm	4 (6.1%)	4 (7.0%)
	Non-shockable rhythm	57 (86.4%)	51 (89.5%)
	Unobtainable	0 (0.0%)	0 (0.0%)
	Unknown	5 (7.6%)	2 (3.5%)
Witness	Unwitnessed	25 (37.9%)	19 (33.3%)
	EMS witnessed	10 (15.2%)	7 (12.3%)
	Bystander witnessed	30 (45.5%)	29 (50.9%)
	Unobtainable	0 (0.0%)	0 (0.0%)
	Unknown	1 (1.5%)	2 (3.5%)
Bystander commenced CPR	Yes	38 (57.6%)	29 (50.9%)
	No	15 (22.7%)	19 (33.3%)
	Not applicable (EMS witnessed)	10 (15.2%)	7 (12.3%)
	Unobtainable	0 (0.0%)	0 (0.0%)
	Unknown	3 (4.5%)	2 (3.5%)

**Table 2:** Concurrent pre-hospital treatments and transfers to hospital.

		Adrenaline N = 66	Placebo N = 57
Intravenous access	Yes	38 (57.6%)	37 (64.9%)
	No	26 (39.4%)	16 (28.1%)
	Unobtainable	0 (0.0%)	0 (0.0%)
	Unknown	2 (3.0%)	4 (7.0%)
Intraosseous access	Yes	33 (50.0%)	24 (42.1%)
	No	31 (47.0%)	30 (52.6%)
	Unobtainable	0 (0.0%)	0 (0.0%)
	Unknown	2 (3.0%)	4 (7.0%)
Time from 999 call to Administration of a trial agent (minute)*	Median (IQR)	22.6 (15.7, 27.2)	21.3 (13.8, 28.7)
	Missing	0	1
Ambulance response time	Median (IQR)	7.5 (4.0, 12.0)	8.0 (4.9, 11.2)
	Missing	0	1
Time for arrival on scene to administration of a trial agent (minute)	Median (IQR)	14.2 (10.4, 18.5)	11.2 (8.5, 16.7)
	Missing	0	1
Administration of Amiodarone	Yes	5 (7.6%)	0 (0.0%)
	No	61 (92.4%)	55 (96.5%)
	Unobtainable	0 (0.0%)	1 (1.8%)
	Unknown	0 (0.0%)	1 (1.8%)
Supraglottic airway used	Yes	46 (69.7%)	38 (66.7%)
	No	18 (27.3%)	15 (26.3%)
	Unobtainable	0 (0.0%)	0 (0.0%)
	Unknown	2 (3.0%)	4 (7.0%)
Endotracheal airway used	Yes	24 (36.4%)	27 (47.4%)
	No	40 (60.6%)	27 (47.4%)
	Unobtainable	0 (0.0%)	0 (0.0%)
	Unknown	2 (3.0%)	3 (5.3%)
Number of shocks after randomisation	Median (IQR)	0 (0, 0)	0 (0, 0)
	Missing	0	1
Patients transported to hospital	Yes	38 (57.6%)	24 (42.1%)
	No	28 (42.4%)	33 (57.9%)
	Unobtainable	0 (0.0%)	0 (0.0%)
	Unknown	0 (0.0%)	0 (0.0%)
Patient declared deceased by Emergency Department staff	Yes	18 (27.3%)	14 (24.6%)
	No	19 (28.8%)	7 (12.3%)
	Not applicable (died on scene)	28 (42.4%)	33 (57.9%)
	Unknown	1 (1.5%)	3 (5.3%)
	Missing	0	0

* Time durations are the difference in minute between two time points. For EMS witnessed cases, the time from 999 call to EMS witness is set to 0 min in all three time interval calculations. ^**^ Patients died on scene.

For the primary outcome a single patient, who was in the adrenaline arm, was alive at 30 days (1/66 (1.5%) compared to 0/57 (0%). However, over three times as many patients achieved a return of spontaneous circulation and were alive at handover to hospital staff (i.e. survived event based on Utstein definition^8^), 16/66 (24.2%) compared to 5/56 (8.9%) unadjusted odds ratio (OR) 3.3 (95% confidence interval 1.1 to 9.6), *p* = 0.03; adjusted OR 5.6 (95% CI 1.6 to 20.4), *p* = 0.009. The sole survivor had an initial rhythm of ventricular fibrillation (VF) rhythm. They had a favourable neurological outcome at hospital discharge which was sustained through to final follow-up at 6 months (Table 3).

**Table 3:** Clinical outcomes.

	Adrenaline	Placebo	Difference (95% Confidence Interval)	Odds ratio (95% Confidence Interval)	
				Unadjusted	Adjusted
Sustained ROSC to hospital admission (n,%)	16/66 (24.2%)	5/56 (8.9%)	15.3% (2.6%, 28.1%)	3.3 (1.1, 9.6), p = 0.03	5.6 (1.6, 20.4), p = 0.009
Survived to hospital discharge	1/66 (1.5%)	0/57 (0%)	1.5% (−1.4%, 4.5%)	2.6 (0.1,67.8), p = 0.6	2.0 (0.3, 15.4), p = 0.5
Favourable neurological outcome at hospital discharge	1/66 (1.5%)	0/57 (0%)	1.5% (−1.4%, 4.5%)	2.6 (0.1,67.8), p = 0.6	2.0 (0.3, 15.4), p = 0.5
Survival to 30 days	1/66 (1.5%)	0/57 (0%)	1.5% (−1.4%, 4.5%)	2.6 (0.1, 67.8), p = 0.6	2.0 (0.3, 15.4), p = 0.5
Survival to 3 months (n,%)	1/66 (1.5%)	0/57 (0%)	1.5% (−1.4%, 4.5%)	2.6 (0.1,67.8), p = 0.6	2.0 (0.3, 15.4), p = 0.5
Favourable neurological outcome at 3 months	1/66 (1.5%)	0/57 (0%)	1.5% (−1.4%, 4.5%)	2.6 (0.1,67.8), p = 0.6	2.0 (0.3, 15.4), p = 0.5
Survival to 6 months (n,%)	1/66 (1.5%)	0/57 (0%)	1.5% (−1.4%, 4.5%)	2.6 (0.1,67.8), p = 0.6	2.0 (0.3, 15.4), p = 0.5
Favourable neurological outcome at 6 months	1/66 (1.5%)	0/57 (0%)	1.5% (−1.4%, 4.5%)	2.6 (0.1,67.8), p = 0.6	2.0 (0.3, 15.4), p = 0.5

Note: Unknown cases are not used in the difference and odds ratio calculation. Adjustment covariates include age, sex (only for sustained ROSC to hospital admission), 999 call to at scene time, at scene to drug administration, initial rhythm (not for sustained ROSC to hospital admission), witnessed event, and bystander CPR. All treatment comparisons are made by comparing treatment Adrenaline arm vs Placebo arm. Firth correction is used to correct the bias caused by small n of events.

## Discussion

In this post-hoc analysis of the PARAMEDIC2 randomised controlled trial, adults who sustained a traumatic cardiac arrest were 3 times more likely to survival to hospital admission if treated with adrenaline, compared to placebo. Long-term survival rates were low, consistent with the findings of others.^9^ The only long-term survivor was in the adrenaline arm.

There is controversy in relation to the optimal approach for managing patients with trauma related out of hospital cardiac arrest. Some guidelines advocate prioritising treatment of reversible causes of cardiac arrest over standard advanced life support guidelines.^2^ In a survey of 16 Emergency Medical Service (EMS) Agencies in the United States who had protocols for traumatic cardiac arrest, three recommended the use of adrenaline whilst three specifically recommended against adrenaline administration. Analysis of 9,565 cases of traumatic out of hospital cardiac arrest recorded in the National EMS Information System found that adrenaline was administered to 60.1% of patients.

The findings of an increase in survival to hospital admission are consistent with the overall findings of PARAMEDIC2,^6^ the Pre-hospital Adrenaline in Cardiac Arrest (PACA)^10^ trial and the Norwegian drug versus no-drug trial.^11^ Non-shockable rhythms are more common in traumatic cardiac arrest, a sub-group known to benefit from adrenaline to a greater degree than those with shockable rhythms.^12^ To our knowledge, this is the only randomised controlled, placebo-controlled trial of adrenaline to report the outcomes of patients with traumatic cardiac arrest.^13^ A systematic review of adrenaline in traumatic out of hospital cardiac arrest identified 4 observational studies with 7158 patients, amongst whom 1909 patients received adrenaline.^14^ Analyses were limited by significant heterogeneity, leading to indeterminate findings with wide confidence intervals for return of spontaneous circulation (OR 4.67, 95% CI 0.66 to 32.81) and in-hospital survival (OR: 0.61, 95% CI 0.11 to 3.37). A Japanese, retrospective observational study explored the time to adrenaline administration amongst 2024 patients who sustained a blunt traumatic cardiac arrest following a road traffic collision. Similar to findings from the PARAMEDIC2 study [19], early adrenaline was associated with improved survival compared to late adrenaline. The degree to which this effect was related to the time of drug administration rather than resuscitation time bias is uncertain.^15^

The Core Outcome Set for Cardiac Arrest (COSCA) prioritises long term survival, neurological outcome and health related quality of life.^16^ However, the relatively small number of traumatic cardiac arrests globally makes conducting trials with sufficient power for longer-term outcomes challenging. A collaboration of clinicians and triallists interested in haemorrhagic shock treated with blood transfusion highlighted similar challenges and advocated the use of earlier outcomes such as 3–6 h survival, to demonstrate efficacy ahead of effectiveness trials.^17^ Taking this approach, the findings of this study could be considered as demonstrating a signal of efficacy for pre-hospital adrenaline in traumatic cardiac arrest.

The findings of this study should be interpreted considering the following limitations. The study was a post-hoc, sub-group analysis of a randomised controlled trial and as such the findings should considered as hypothesis generating rather than definitive evidence of cause and effect. The number of patients included in the analysis was small, limiting the power to identify differences in longer term outcomes. It is usual to report the adjusted analyses as our main findings. However, we found that due to the small number of patients, making the adjustments for all the same covariates was challenging. Thus, different adjustments were made for different outcomes. The trial did not collect information on the injuries sustained leading to traumatic cardiac arrest or the use of adjunctive treatments (e.g. thoracostomy, thoracotomy) which may have introduced confounding, selection and / or performance bias.

## Conclusion

Adrenaline was associated with increasing survival to hospital admission compared to placebo. These data support the use of adrenaline in trauma related out of hospital cardiac arrest.

## CRediT authorship contribution statement

**C. Ji:** Writing – original draft, Investigation, Funding acquisition, Formal analysis, Data curation, Conceptualization. **H. Pocock:** Writing – review & editing, Methodology, Data curation, Conceptualization. **C.D. Deakin:** Writing – review & editing, Methodology, Investigation, Funding acquisition, Data curation, Conceptualization. **T. Quinn:** Writing – review & editing, Methodology, Funding acquisition, Data curation, Conceptualization. **J.P. Nolan:** Writing – review & editing, Methodology, Investigation, Funding acquisition, Data curation, Conceptualization. **N. Rees:** Writing – review & editing, Investigation, Data curation, Conceptualization. **K. Charlton:** Writing – review & editing, Investigation, Data curation, Conceptualization. **J. Finn:** Writing – review & editing, Methodology, Funding acquisition, Conceptualization. **A. Rosser:** Writing – review & editing, Investigation, Data curation, Conceptualization. **R. Lall:** Writing – original draft, Supervision, Methodology, Investigation, Funding acquisition, Formal analysis, Data curation, Conceptualization. **G.D. Perkins:** Writing – review & editing, Writing – original draft, Supervision, Project administration, Methodology, Investigation, Funding acquisition, Conceptualization.

## Declaration of competing interest

The authors declare the following financial interests/personal relationships which may be considered as potential competing interests:

GDP is Editor in Chief of Resuscitation Plus. JPN is an editorial board member of Resuscitation Plus. The article was handled and all editorial decisions were taken by an independent editor.

The authors declare receiving funding from the National Institute for Research. NR additionally received support from Health Care Research Wales.
