# Supplementary material for: Adrenaline for traumatic cardiac arrest: A post hoc analysis of the PARAMEDIC2 trial
Source: Resusc Plus. 2025 Feb 4;22:100890. doi: 10.1016/j.resplu.2025.100890 (PMC11872399; doi:10.1016/j.resplu.2025.100890)
Supplement: Supplementary Data 1 [file mmc1.docx]

**Supplementary material**

**Adrenaline for traumatic cardiac arrest: a post hoc analysis of the PARAMEDIC2 trial**

C Ji et al

List of covariates included in the adjusted statistical analyses.

| Covariate | Definition | Model Adjustments made for sustained ROSC to hospital admission | Model Adjustments made for Survival and neurological outcomes post hospital admission |
| --- | --- | --- | --- |
| Age | Age in years at cardiac arrest | Yes | Yes |
| Sex | Male, female | Yes | No* |
| Ambulance response time | Time (minutes) from randomisation to EMS arrival on scene | Yes | Yes |
| Time for arrival on scene to administration of a trial agent | Time (minutes) from EMS arrival on scene to administration of a trial agent | Yes | Yes |
| Initial cardiac rhythm | Asystole, VF, Pulseless VT, PEA, Bradycardia, AED nonshockable, AED shockable | No* | Yes |
| Whether the cardiac arrest was witnessed | Unwitnessed, EMS witnessed, Bystander witnessed | Yes | Yes |
| Whether CPR was performed by a bystander | Yes, no | Yes | Yes |

* Variable is not adjusted for to avoid model convergence failure.

Analysis of small samples often comes with complete or quasi separation problem,[1] leading to biased estimation using the standard maximum likelihood method. Covariates were removed from the appropriate models, to avoid the quasi separation problem, for all models. For the survival and neurological outcomes post hospital admission models, the Firth’s method was further applied, This corrects the maximum likelihood estimation to reduce the small sample bias using a score function.[2]

**References**

[1] Heinze G., Schemper M. (2002). A solution to the problem of separation in logistic regression. Stat. Med. 21, 2409–2419 10.1002/sim.1047

[2] Firth, D. (1993): "Bias reduction of maximum likelihood estimates", Biometrika 80(1): 27-38; (doi:10.1093/biomet/80.1.27)
